# Kicking the mental number line: a kinematic investigation of numerical processing in childhood

**DOI:** 10.3389/fpsyg.2026.1765058

**Published:** 2026-02-25

**Authors:** Elisa Straulino, Silvia Benavides-Varela, Giovanni Bruno, Luisa Sartori, Rosa Rugani

**Affiliations:** 1 Department of General Psychology, University of Padova, Padova, Italy; 2 Department of Developmental Psychology and Socialization, University of Padova, Padova, Italy; 3 Padova Neuroscience Center, University of Padova, Padova, Italy

**Keywords:** action execution, childhood, kinematics, number processing, numerical cognition

## Abstract

A growing body of evidence in adults indicates bidirectional interactions between numerical processing and motor execution, often interpreted as the result of sensorimotor experiences linking numerical magnitude to physical size and action parameters. However, the developmental trajectory of number–action coupling, particularly for non-symbolic numerosities, remains largely unexplored. In the present study, we provide a developmental extension of a kinematic paradigm originally introduced in adults to investigate whether observing non-symbolic numerical stimuli modulates motor performance in preschool (4–6 years) and school-age (7–11 years) children. Using an engaging finger–kick task, we measured fine-grained kinematic parameters while children responded to small (2-Dots) or large (8-Dots) dot arrays, as well as to a non-numerical baseline stimulus. Preschoolers exhibited more efficient movement trajectories after observing small numerosities compared to the baseline condition, together with greater leftward trajectory deviations—consistent with a spatial–numerical mapping aligned with the Mental Number Line. In contrast, no kinematic modulation was observed following exposure to large numerosities, and no reliable numerical effects emerged in school-age children, mirroring patterns previously reported in adults. Taken together, these findings suggest that non-symbolic spatial–numerical mappings can directly influence action execution early in development, but may be reshaped or attenuated with increasing experience and formal education. By combining non-symbolic number processing with precise kinematic measures in children, the present study sheds light on early number–action links and their developmental reorganization, offering insights into the foundations of numerical cognition.

## Introduction

Humans share with many animal species a primitive, non-verbal number system capable of encoding the numerosity of objects in space and events in time (Butterworth, 1999; Dehaene, 2011; Rugani, 2018). This number sense supports rapid but approximate estimation across space, time, modality, and format (Arrighi et al., 2014; Burr et al., 2017; Burr and Ross, 2008; Togoli et al., 2021). According to ATOM (A Theory of Magnitude), space, time, and number rely on shared neural metrics and substrates, enabling coordinated perception and action (Walsh, 2003). These links are evident in everyday motor behavior—such as grasping—where the brain simultaneously computes object size, hand aperture, and distance. This tight coupling between magnitude and action has led to the proposal of sensorimotor number systems, integrating numerical and motor processes (Anobile et al., 2014, 2022; Cordes et al., 2001; Feigenson et al., 2004; Kirschhock and Nieder, 2022; Ross, 2003; Whalen et al., 1999).

The propensity to spatially represent environmental information is a core characteristic of the human cognitive system (Gevers et al., 2003). Numbers, in particular, are systematically coded into space along a left–right oriented continuum (Fias and Fischer, 2005; Bueti and Walsh, 2009; Dehaene, 2011). The seminal intuition about this spatial-numerical association dates back to Galton (1880), who first proposed that humans mentally arrange numbers along the Mental Number Line (MNL) increasing from left to right, with small numbers on the left and large numbers on the right. More than a century later, the first empirical demonstration of this spatial mapping was provided by Dehaene et al. (1993), who showed that people respond faster to smaller numbers on the left and to larger numbers on the right, an association known as the Spatial-Numerical Association of Response Codes (SNARC) effect.

Despite this robust spatial grounding of numbers, research on how numerical and motor systems interact has been conducted mostly in adults. A substantial body of evidence shows bidirectional interactions between symbolic numbers and hand movements (Andres et al., 2004; Lindemann et al., 2008; Perrone et al., 2010; Gianelli et al., 2012; Namdar et al., 2014). On the one hand, executing specific actions modulates numerical processing. For instance, grip closing slows the processing of large numbers, whereas pointing disrupts the distance effect for small ones, suggesting that different hand actions selectively interfere with numerical evaluation (Badets and Pesenti, 2010; Ranzini et al., 2022). On the other hand, numerical processing shapes motor behavior. Small digits facilitate grip closure, whereas large digits facilitate grip opening (Andres et al., 2004). Large numbers also elicit wider grip apertures (Lindemann et al., 2008), and modulate the kinematics of object manipulation (Gianelli et al., 2012) and action estimation (Badets et al., 2007). These findings converge on the idea that small numbers align with hand-closing actions and large numbers with opening actions—a mapping likely shaped by everyday experience (e.g., small objects require small apertures) and therefore difficult to disentangle from learned sensorimotor associations. Grasping movements also undergo age-related developmental changes (Kuhtz-Buschbeck et al., 1998; Zorzi et al., 2002), further complicating developmental investigations.

To overcome these limitations, Rugani et al. (2017, 2018) introduced a kinematic paradigm involving a finger-based kick executed within a 3-D motion capture setup. The present study builds on this paradigm as a developmental and translational extension. Unlike grasping, this movement reduces experiential biases and developmental confounds, making it particularly suitable for investigating number–action coupling across age groups. In this task, adults exhibited clear numerical effects: they kicked leftward more often after seeing small numbers, and their movement trajectories were more efficient after viewing large symbolic digits.

Evidence for spatial–numerical associations with non-symbolic stimuli in adults is mixed. While some studies report reliable behavioral and kinematic effects (e.g., Nuerk et al., 2005; Nemeh et al., 2018), others fail to observe consistent signatures (e.g., Prpic et al., 2023). This variability likely reflects differences in task structure and stimulus properties, as well as the pervasive influence of symbolic number knowledge and culturally shaped spatial conventions. Years of experience with symbolic numbers may modulate, mask, or override more basic non-symbolic mappings, potentially accounting for null effects reported in adult samples (Rugani et al., 2017).

Investigating children before—and during—the acquisition of symbolic number knowledge is therefore theoretically informative, as it provides access to pre-symbolic numerical representations (De Hevia et al., 2017; Di Giorgio et al., 2019). Developmental and comparative evidence consistently demonstrates spatial biases for non-symbolic numerosities even when continuous physical variables are controlled (e.g., De Hevia and Spelke, 2009; Rugani et al., 2015; Giurfa et al., 2022; Regolin et al., 2025), suggesting an early link between number and space that may be reshaped across development.

### The developmental gap

Despite extensive evidence in adults, the developmental trajectory of number–action coupling remains underexplored. Emerging findings suggest that numerical–motor associations may arise early in life (Decarli et al., 2025). Hand movements appear to scaffold the acquisition of numerical concepts (Novack et al., 2014; Fischer et al., 2015), supporting the hypothesis that the integration of number and action plays a foundational role during childhood (Rusconi et al., 2006; Rugani and Sartori, 2016). Neurocognitive evidence reinforces this claim: parietal regions implicated in numerosity processing also support spatial and motor functions, and infants as young as 3–9 months show sensitivity to congruency between numerosities and hand shapes (Decarli et al., 2022a, 2022b).

These findings support the existence of an early, preverbal number–action mapping, potentially grounded in the embodied role of fingers in numerical development (Sixtus et al., 2023; Krenger and Thevenot, 2024; Orrantia et al., 2024; Roesch et al., 2024; Soylu, 2024; Fu and He, 2025). Notably, the Latin term *digit* — for both fingers and written numbers — echoes this deep historical and cognitive link.

The present study extends the kinematic paradigm of Rugani et al. (2018) to preschool and school-age children to investigate how non-symbolic numerosity influences motor execution during development. Children wore a miniature soccer shoe on their right index finger and kicked a small ball toward a frontal goal after viewing numerical (2- or 8-Dots arrays) or baseline non-numerical stimuli (a soccer goal). A no-go signal (5-Dots) ensured numerical processing. Movements were recorded with a 3-D motion capture system. Because the motor response was identical in all trials, any systematic kinematic variation can be attributed to numerical processing.

We hypothesized that if the spatially-oriented mental number line influences motor execution, children would exhibit greater leftward deviation after seeing small numerosities (2-Dots) relative to baseline, but not after seeing large ones (8-Dots). Large numbers, linked to right space, would either produce no deviation or a rightward shift. However, as stated above, in this paradigm, rightward effects are weaker or absent.

By combining number presentation with precise action measurement in children, this study aims to shed light on early number–action mappings and on how these mappings are preserved, reshaped, or reweighted across development, distinguishing foundational cognitive mechanisms from those increasingly shaped by formal education and experience.

## Method

All experiments were conducted in accordance with the Declaration of Helsinki and approved by the Ethics Committee of the University of Padova (protocol No. 4782). All parents gave written informed consent prior to participation. All participants were naïve to the purposes of the experiment.

### Participants

Twenty-one right-handed children (7 females and 14 males, mean age = 8 years, age range: 4 years and 10 months – 11 years and 10 months) took part in the experiment. Handedness was assessed with a brief questionnaire asking participants which hand they used to grasp a pen, for coloring, and for eating. Concordance was assessed by observing which hand the participant used to insert their index finger into the soccer shoe during the test. Sample size was determined by means of GPOWER 3.1 (Erdfelder et al., 1996) based on previous literature (Rugani et al., 2018). Since we used a repeated-measures ANOVA, we considered an effect size f of 0.3, alpha = 0.05, and power = 0.8. The projected sample size needed with this effect size was *N* = 20 for within-group comparisons.

To better investigate the relationship between number and movement during the critical age for the gradual acquisition of math skills, we explored the data by dividing the group into two clusters. A cluster of preschool children who had not received formal math education and a cluster of school-age children with corresponding academic math skills. Mathematical abilities were assessed with a Mathematical Proficiency Index, MPI, based on three scales: (i) Enumeration Time (ET) from 1 to 20 in 20″ (expressed as the total time, 20″, minus the time taken to pronounce the entire list without errors), (ii) Additive Calculation Ability (ACA) in 30″ (expressed as the number of additions performed correctly out of a total of 20 in the time interval), (iii) Subtractive Calculation Ability (SCA) in 30″ (expressed as the number of subtractions performed correctly out of a total of 20 in the time interval; see Supplementary Table S1 for the list of demanding math tasks). Synthetically, the formula for calculating the Math Proficiency Index is:

$$\mathrm{MPI}=\Sigma \;[(20^{'}-\mathrm{ET})+\mathrm{ACA}+\mathrm{SCA}]$$

Once it was verified that none of the children in the pre-school group had properly mastered the ability to count, add, or subtract and none reached the criterion – while the school-aged children had an average score (MPI = 33) in the test range (0–60) – we divided the sample into two subgroups accordingly. The resulting clusters were therefore: (i) a Preschool group with no formal math education (4 males and 3 females, mean age = 5 years and 8 months, age range: 4 years and 10 months – 6 years and 10 months); (ii) a group of school-aged children (10 males and 4 females, mean age = 9 years and 1 month, age range: 7 years and 6 months – 10 years and 10 months). All participants had normal or corrected-to-normal vision and were naive about the purpose of the experiment. The data obtained from the school-age participants in this test are shown in Table 1.

**Table 1:** Mathematical proficiency index of the schoolchildren.

Participant age (months)	Enumeration time – ET (range 0–20)	Additive calculation ability – ACA (range 0–20)	Subtractive calculation ability – SCA (range 0–20)	Mathematical proficiency index – MPI (range 0–60)
90	1	10	4	15
102	8	11	4	23
107	9	10	5	24
110	9	11	4	24
105	5	11	9	25
129	4	13	11	28
121	4	15	11	30
121	5	15	17	37
126	6	18	14	38
110	10	14	15	39
90	8	17	15	40
102	5	16	20	41
97	12	20	10	42
101	12	17	14	43

### Apparatus

Participants were tested individually in a dimly lit room. The stimuli presentation was implemented using E-prime V2.0. One infrared reflective marker (ultra-light 6 mm diameter semi-spheres) was applied to the right index finger for kinematic analysis. A further marker was placed on the ball to perform a detailed analysis of the deviation movements of the index with respect to this reference point. Six infrared cameras (sampling rate: 70 Hz, adequate to record spatial and low-frequency temporal parameters) were placed in a semicircle at a distance of 1–1.2 m from the center of the room captured the relative position of the markers. The movements were recorded using a 3-D motion analysis system (SMART-D, Bioengineering Technology and Systems [B|T|S] BTS, Milano, Italy). Prior to data collection, the image captured by each camera was optimized by adjusting its zoom, focus, threshold and brightness. The system was then calibrated in order to define an absolute spatial reference system. For the static calibration, a three-axes frame of 5 markers at known distances from each other was placed in the middle of the table. For the dynamic calibration, a three markers wand was moved throughout the workspace of interest for 120 s. The spatial resolution of the recording system was 0.2 mm over the field of view. The standard deviation of the reconstruction error was 0.2 mm for the x, y and z axes.

### Stimuli

The experimental stimuli were matrices of non-symbolic numbers: 10 different con**fig**urations of two (2-Dots) and eight dots (8-Dots). These numerosities were selected for methodological consistency with prior work and because they are reliably discriminable (Rugani et al., 2017). The dots were black with a diameter of 2 cm and were arranged in a white square of 964×964 pixels (see Supplementary Table S2 for stimulus properties). The arrangement of the dots was such as to avoid both a clumping effect if they were too close together and a scattering effect if they were not (Rugani et al., 2017). The program used to create the stimuli was GeNEsIs, a tool for generating controlled stimuli in non-symbolic numerical experiments (Zanon et al., 2022). A non-numerical stimulus (drawing of a soccer goal) was presented during baseline trials. This picture was a salient stimulus capable of sustaining children’s motivation and attention. It was perfectly symmetrical, thus avoiding any indication of direction. A stimulus with five dots (5-Dots) positioned according to the standard arrangement of a die was adopted as a “no-go” signal to ensure that movements did not occur automatically without cognitively processing the numerosity of the stimulus.

### Procedure

**Figure 1:**
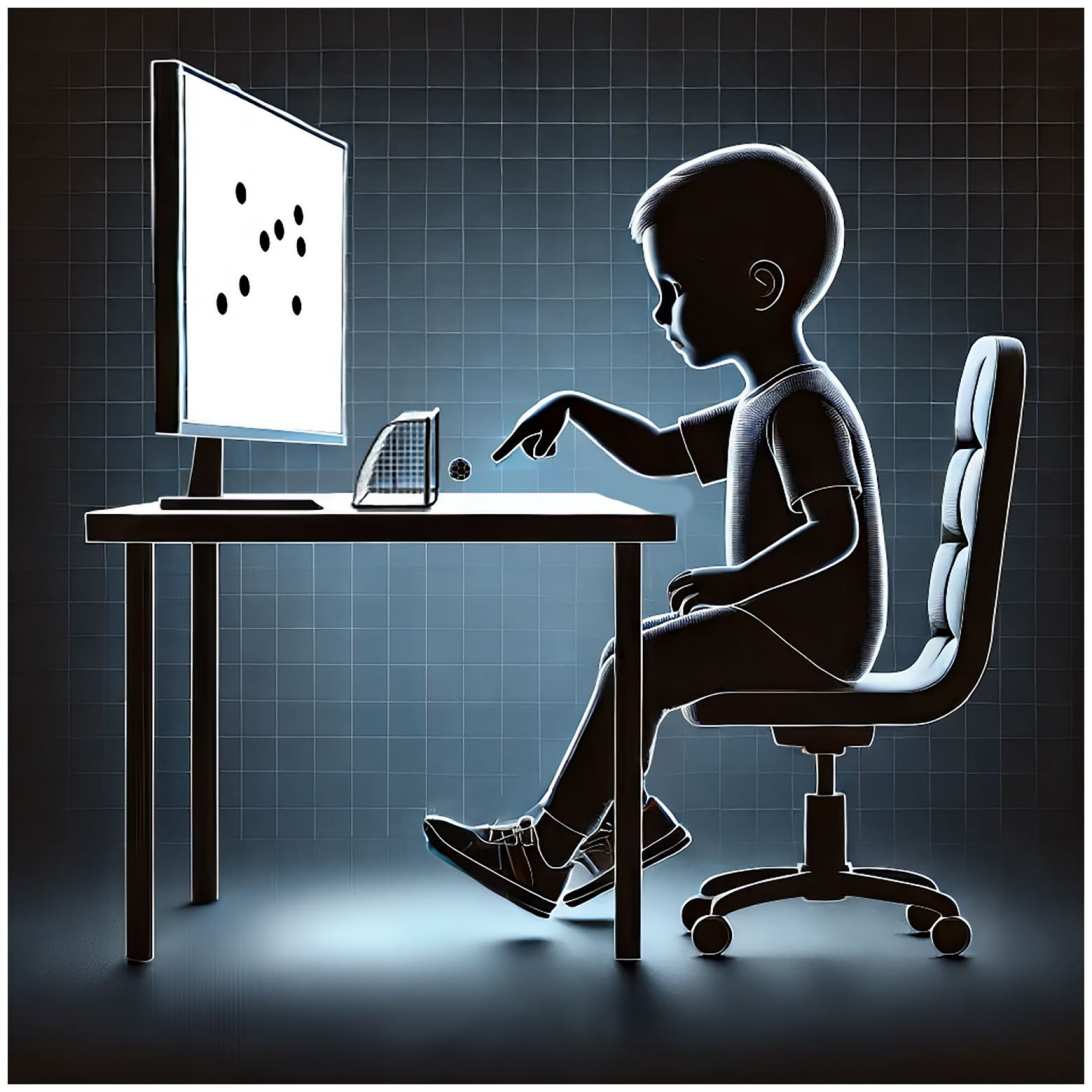
Illustration of the experimental setup. Participants were seated in front of a monitor that displayed an experimental stimulus. A small soccer goal was placed centrally to the participant’s position. A 3D-optoelectronic SMART-D system was used to track the kinematics of the participant’s right index finger and the position of the ball by means of six cameras and two infrared reflective markers attached to the participant’s index finger and the ball. Participants were instructed to kick the ball toward the soccer goal at their own pace, when a black fixation cross disappeared. During the testing session, participants kicked the ball upon random presentation of either 2-dots or 8-dots. Figure created with generative AI and modified by the authors.

We capitalized on a previous experimental paradigm developed in our laboratory and applied so far only to the adult population (Rugani et al., 2018). Participants sat on a chair in front of a table with their right hand located in the designated start position. Participants’ right index was introduced in a small plastic soccer shoe (3 cm long, 1.5 cm wide) positioned on a footprint. In the start position, participants rested their right wrist on a pillow (16 cm long, 11 cm wide and 6.5 cm high), which was shaped to guarantee a comfortable and repeatable posture of the hand, allowing them to effortlessly kick the ball. A plastic ball (2.3 cm in diameter) was located at 1 cm and a soccer goal (18 cm long, 16 cm high) at 50 cm away from the footprint. Given the novelty of the task, a training phase was implemented so that children could practice or adjust the soccer shoe until they felt comfortable with kicking the ball with their finger. Moreover, in order to increase their motivation towards the task, during familiarization they were shown a sketch of the FIFA soccer video game and told they were about to play a similar kicking game, where dots represented opposing players to be kicked against. A monitor set at eye level (the eye–screen distance was 80 cm) was used to present the experimental stimuli (Figure 1). Participants underwent two sessions (i.e., Baseline and Testing) and were instructed to kick the ball toward the soccer goal following the stimulus presentation, at their own pace. No instruction was given concerning the speed of movement. A black fixation cross appeared for 100 ms and was replaced with a stimulus after 1,000 ms. There were 40 trials in total. The Baseline session was presented for 10 trials. During the Testing session participants kicked the ball upon random presentation of either 2-Dots or 8-Dots (10 trials each). On each trial, after kicking the ball, children were requested to say aloud if they saw a large or a small number of “opponents” (dots). This was meant to ensure children were paying attention to the numerical stimuli and not only focused on the movement itself. In addition, whenever 5-Dots were presented (*n* = 10 trials), participants were required to refrain from kicking the ball, which prevented participants from falling into a routine response pattern and maintained engagement throughout the session. The configuration of the 5-Dots stimuli was always the same (dice-like).

### Kinematic analysis

After data acquisition, the 3-D coordinates of the markers were pre-processed using the SMART-D Tracker (BTS) software to reconstruct the trajectories of the index finger. Subsequently, the SMART-D Analyzer (BTS) software was used to filter and analyze the data. The analysis protocol was specifically designed to extract spatial, velocity, and temporal dependent variables. First, signal frequencies above 10 Hz were eliminated using a low-pass filter. Next, key events or time instants were identified in the time traces of distance, velocity (calculated as the derivative of distance), and deceleration (the derivative of velocity). The onset and offset of the movement were determined from the velocity trace of the index finger marker. Specifically, movement onset was defined as the moment when velocity exceeded a threshold of 5 mm/s and remained above this value for at least 500 ms. Movement offset was defined as the moment when the tangential velocity of the finger marker dropped below 5 mm/s after the ball was kicked. To account for inter-individual and inter-trial variability in movement duration, temporal variables were normalized to the total movement time (MT) by dividing the absolute values by MT. The resulting normalized value thus represents the proportion of the movement duration at which each event occurred, with values ranging from 0 to 1. Following the methodology of Rugani et al. (2018), the following spatial, velocity, and temporal parameters were calculated:

#### Spatial parameters (mm)

Maximum Trajectory Height (MH): the maximum value of the Y-coordinates of the index.

Maximum Deviation – Right (MDevRight): the maximum value of the perpendicular right deviation of the index finger from the virtual line linking the starting position with the target object.

Maximum Deviation – Left (MDevLeft): the maximum value of the perpendicular left deviation of the index finger from the virtual line linking the starting position with the target object.

#### Velocity parameters (mm/s and mm/s^2^)

Maximum Velocity (MV): the maximum value of index finger 3-D velocity.

Maximum Deceleration (MDec): the absolute value of the maximum rate at which the index finger slowed down.

#### Temporal parameters (ms and %)

*Movement Time (MT): the total duration of the movement, from onset to offset.*

Time to Maximum Velocity (TMV): the time at which index velocity was maximum, normalised with respect to MT.

Time to Maximum Deceleration (TMDec): the time at which index deceleration was maximum, normalized with respect to MT.

Time to Maximum Deviation – Right (TMDevRight): the time at which trajectory reached the maximum perpendicular right deviation from the virtual line linking the starting position with the target object, normalized with respect to MT.

Time to Maximum Deviation – Left (TMDevLeft): the time at which trajectory reached the maximum perpendicular left deviation from the virtual line linking the starting position with the target object, normalised with respect to MT.

### Data analysis

**Figure 2:**
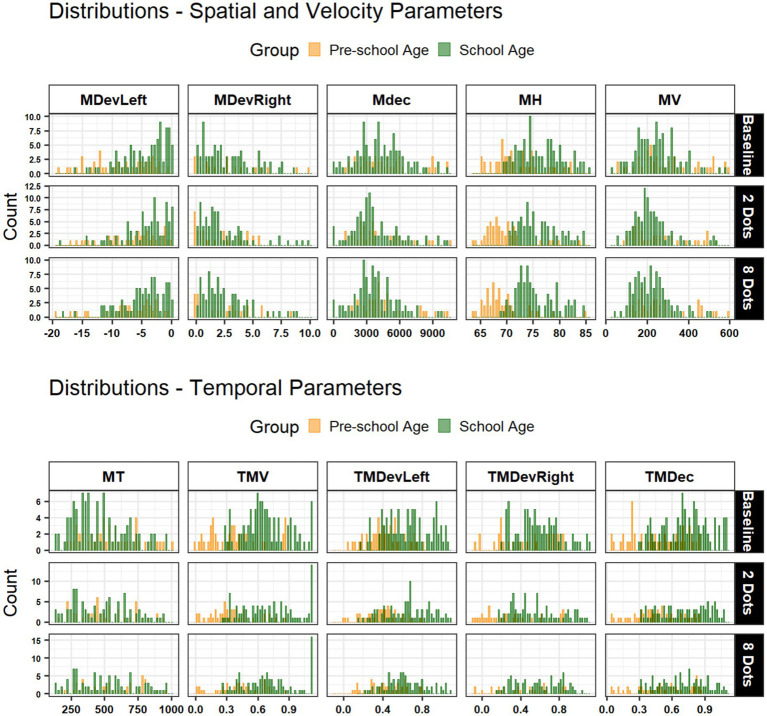
Distributions of spatial, velocity, and temporal parameters across groups. Histograms show the distribution of parameter values for pre-school age (orange) and school age (green) participants, separately for conditions (vertical facets, columns) and type of stimulus (baseline, 2-Dots, and 8-Dots; horizontal facets, rows). Parameters are grouped into spatial and velocity parameters (top panel: MDevLeft, MDevRight, MDec, MH, MV) and temporal parameters (bottom panel: MT, TMV, TMDevLeft, TMDevRight, TMDec).

**Figure 3:**
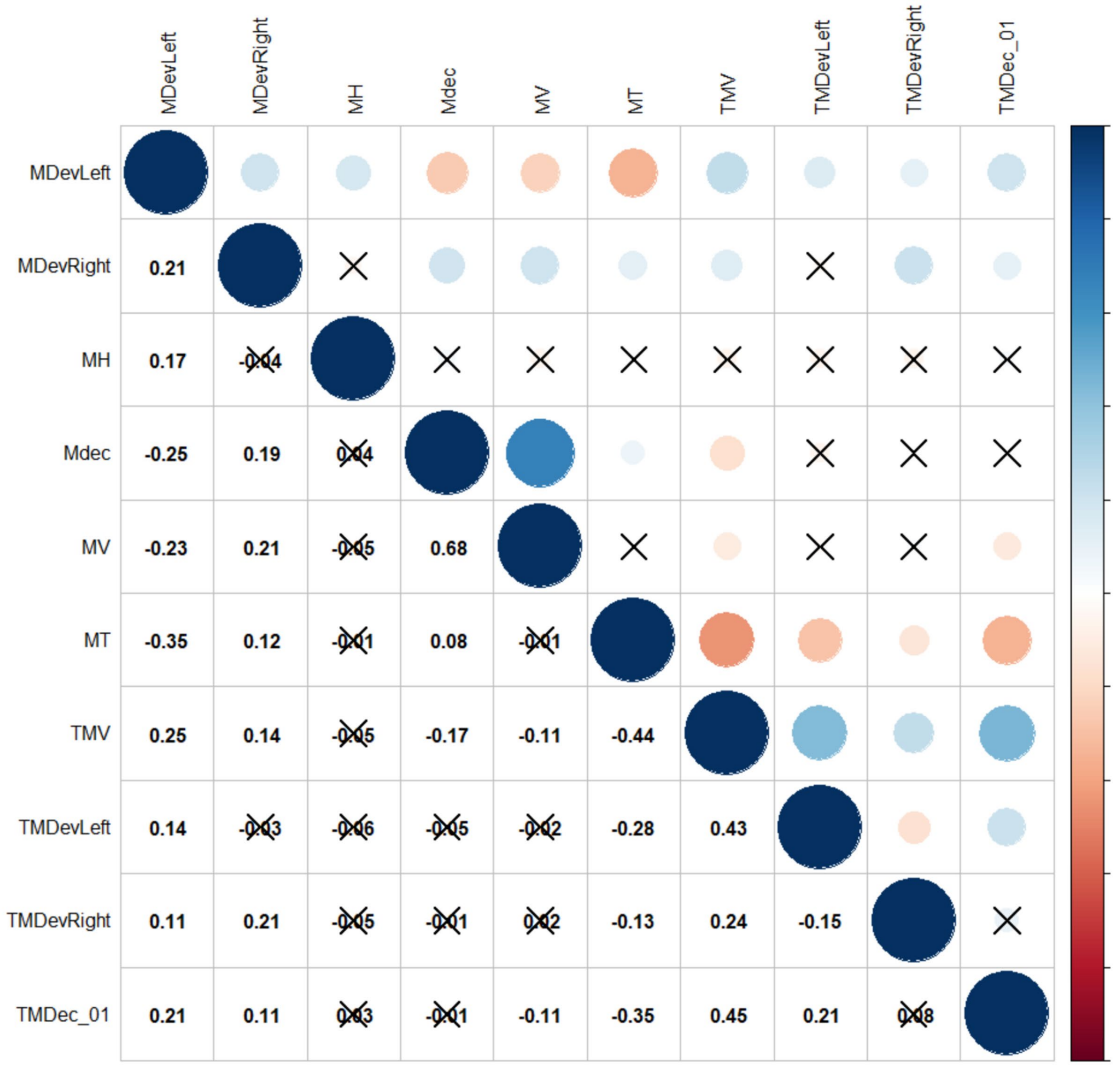
Pearson’s pairwise correlations among kinematic parameters. In each correlation cell of the upper panel, the size and color of the circles represent the strength and direction of the correlations (blue = positive, red = negative), while, in the lower panel, correlation scores are reported. Crosses indicate non-significant correlations (Bonferroni-corrected).

Children performed the verbal magnitude judgment task with 100% accuracy, and no-go trials showed fewer than 2% errors. Data analyses were performed in the R environment (R Core Team, 2025). In the data cleaning phase, the 10 kinematic parameters of interest were standardized in z-scores and data were filtered by excluding outliers’ values greater than ±3 SD from the mean, to avoid their potential influence. Overall, less than 5% of the total data were excluded. The final dataset was composed of 576 observations from 21 individuals. Descriptive and inferential statistical investigations were performed on all the 10 kinematic parameters of interest to investigate possible differences in performance between groups (Pre-schoolers as reference level, Schoolers) when observing the numerical stimulus “2-Dots” (as reference level) compared to stimulus “8-Dots” and the baseline (no dots). Figure 2 describes the distribution of each parameter controlling for Group and Stimulus (2, 8-Dots). Pairwise Pearson correlations among the variables of interest were computed (Figure 3), and Bonferroni corrections were applied (corrplot R package; Wei and Simko, 2017). Finally, data were analyzed using generalized linear mixed models (GLMMs) implemented in the glmmTMB R package (Brooks et al., 2017). This approach was chosen for its flexibility in modelling several non-Gaussian outcomes, including beta- and gamma-distributed dependent variables, and for its ability to accommodate random effects structures. Models – one per each kinematic parameter as dependent variable - included fixed effects for Group, Stimulus, and their interaction, with a random intercept for Participant to account for repeated observations. All the models considered the 2-Dots experimental condition as reference level. Although our sample size was limited due to practical constraints, the use of glmmTMB R package and GLMMs is suitable for small to moderate samples and allows flexible specification of distributions while accounting for participant-level random effects. To mitigate small-sample limitations, we reported marginal and conditional R2 (Nakagawa and Schielzeth, 2013) and inspected model fit with DHARMa diagnostics (Hartig, 2022). *Post hoc* contrasts were conducted on estimated marginal means using emmeans (Lenth, 2024), with *p*-values adjusted for multiple comparisons. Distribution test on each variable was then performed to determine the reference family distribution to apply in each model (fitdistrplus), and potential alternative distributions were compared using goodness-of-fit statistics provided by the same R package (‘gofstat’ function). Considering the evidence collected and the residual check from each model, in the Result section are presented results from five models which resulted of more interest (m1: MH; m2: MDevLeft; m3: MV; m4: TMDevRight; m5: TMV). Additional descriptives are provided in the Supplementary Figure S1 and Supplementary Table S3. The dataset and the R script are available in the OSF project folder: https://osf.io/unf9y/?view_only=780672a28ec947ba9fa469308c425ace.

## Results

The distribution of kinematic parameters controlled by Group (Pre-school, School age) and Stimulus (Baseline, 2 and 8-Dots) is shown in Figure 2. The magnitude and direction of parameter scores and overall distribution highlight Group differences in terms of overlap. Interesting insights come from Maximum Trajectory Height (MH) distribution, which evidence a clear separation between preschoolers and school-aged children.

Additional insights come from the relationship between kinematic parameters. Figure 3 depicts their pairwise correlations, corrected for the number of comparisons. Overall, all kinematic parameters move consistently, showing high internal correlations between time measurements (MT, TMV, TMDevLeft, TMDevRight, TMDec) and speed measurements (MV, MDec). In particular, it is interesting to note in both groups a consistent anticipation of all peaks (TMV, TMDevLeft, TMDevRight, TMDec) as movement time (MT) increases, confirming that reaching coordination is already mastered at an early age. The anticipation of peaks is in fact preparatory to achieving greater accuracy during the finalization phase of movement (Castiello, 2005).

A crucial finding is the almost total absence of correlations between Maximum Trajectory Height (MH) and the rest of the parameters (with the exception of Maximum Deviation – Left, MDevLeft), indicating that this measure stands apart from the group, and will be further investigated.

As previously mentioned, all models included the interaction between Group (Pre-school vs. School age) and Stimulus condition (2-Dots, 8-Dots, Baseline) as fixed effects, while controlling for variability across participants by including a random intercept for Participant (see Table 2 for mean values).

**Table 2:** Kinematic parameters (mean and standard deviation).

Parameters	2-Dots	8-Dots	Baseline
MH	73.646 ± 4.820	73.666 ± 5.037	74.688 ± 4.526
MDevLeft	−4.547 ± 4.077	−4.944 ± 4.061	−5.382 ± 4.600
MDevRight	2.333 ± 2.256	2.547 ± 2.181	2.912 ± 2.514
MV	224.495 ± 101.091	229.873 ± 95.126	251.564 ± 110.528
MDec	3767.097 ± 2183.816	3881.817 ± 2021.439	4230.518 ± 2261.298
MT	458.218 ± 210.510	469.266 ± 225.242	443.697 ± 210.776
TMV	0.614 ± 0.260	0.633 ± 0.249	0.620 ± 0.250
TMDec	0.673 ± 0.258	0.683 ± 0.265	0.690 ± 0.259
TMDevRight	0.443 ± 0.265	0.499 ± 0.249	0.511 ± 0.245
TMDevLeft	0.543 ± 0.227	0.561 ± 0.215	0.549 ± 0.225

Model 1 (m1, Supplementary Table S4) investigated the impact of Group and Stimulus condition on the dependent variable MH, modelled with a t-distribution. Results indicated a significant main effect of Group, with School-age children showing higher MH values compared to Pre-schoolers in 2-Dots stimuli (*b* = 6.58, SE = 1.40, *z* = 4.70, *p* < 0.001), likely due to finger anatomy. A significant main effect of Stimulus also emerged at the Group reference level (Pre-schoolers), showing significant comparison between 2-Dots and Baseline (*b* = −1.11, SE = 0.18, *z* = −6.01, *p* < 0.001) and between 2-Dots and 8-Dots (*b* = 0.50, SE = 0.16, *z* = 3.09, *p* = 0.002). Importantly, a significant Group × Stimulus interaction was observed. To further explore it, post-hoc pairwise comparisons (Bonferroni-corrected) were conducted within each group. Pre-schoolers performed lower trajectories for the 2-Dots condition than for the Baseline condition (estimate = −1.72, SE = 0.31, *z* = −5.52, *p* < 0.001). No significant difference was found between the 2-Dots and 8-Dots conditions (*p* = 1.00). For the Schoolers, neither the comparison between 2-Dots and Baseline (*p* = 0.120) nor between 2-Dots and 8-Dots (*p* = 1.00) reached significance. Overall, these results suggest that the stimulus manipulation primarily affected performance in the Pre-schoolers, while School-age children showed more stable MH trajectories across stimulus conditions.

**Figure 4:**
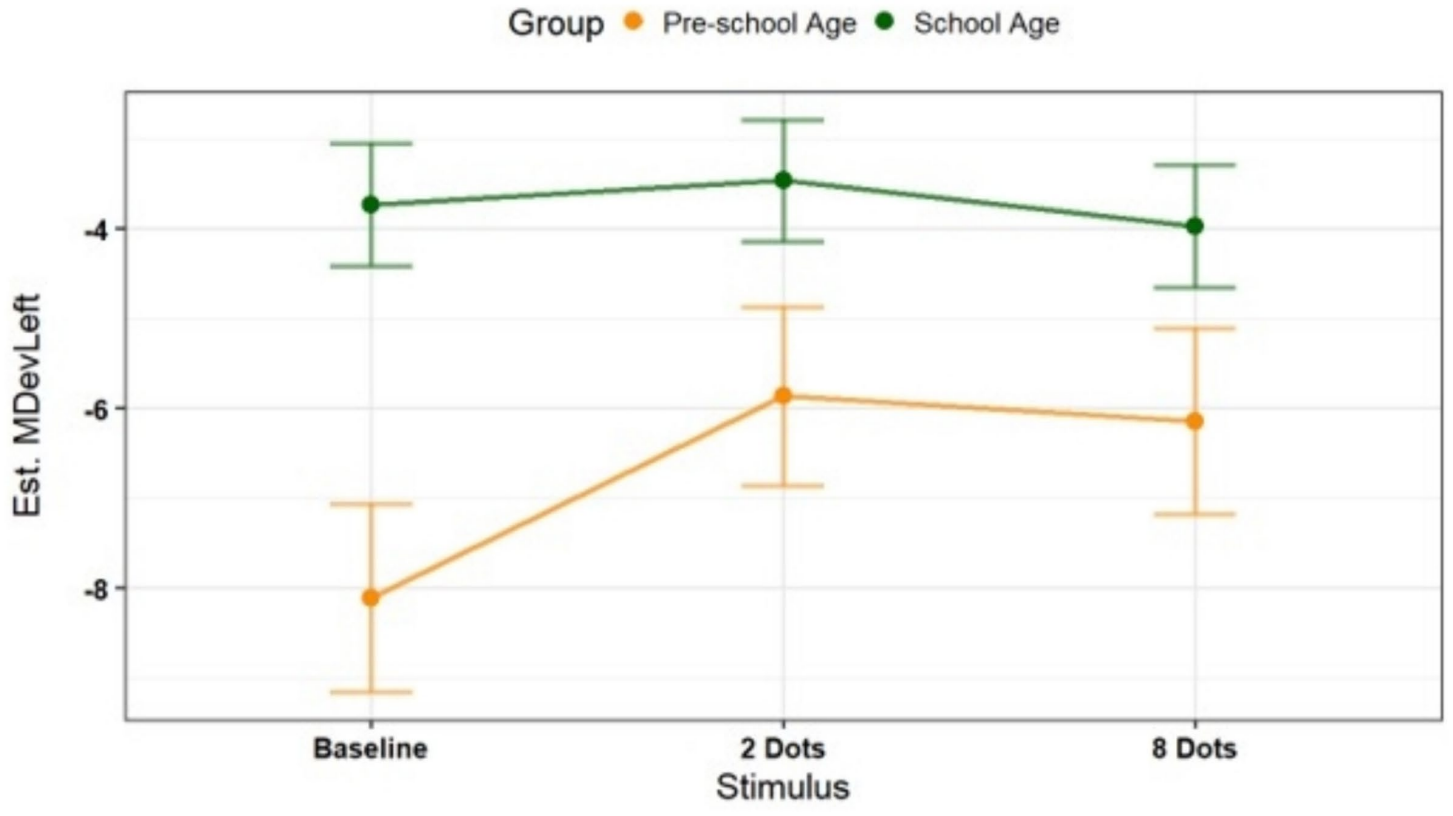
Interaction effect on maximum deviation – left. The graph shows a significant Group × Stimulus interaction in the leftward deviation parameter. Only pre-schoolers performed greater leftward deviations for the 2-dots condition than for the baseline condition.

Model 2 (m2, Supplementary Table S5) investigated the impact of Group and Stimulus condition on the dependent variable MDevLeft, modelled with a t-distribution. Also in this case, significant main effects of the two predictors were detected. A significant main effect of Group was found, with School-age children showing higher MDevLeft values compared to Pre-schoolers at the stimulus reference level (*b* = 2.98, SE = 1.13, *z* = 2.64, *p* = 0.008), likely due to finger anatomy. A significant main effect of Stimulus also emerged, showing significant comparison between 2-Dots and Baseline in Pre-schoolers (*b* = 1.40, SE = 0.45, *z* = 3.13, *p* = 0.002), while no significant difference was observed with the 8-Dots condition. A significant Group × Stimulus interaction was observed. Pre-schoolers performed greater leftward deviations for the 2-Dots condition than for the Baseline condition (estimate = 2.24, SE = 0.72, *z* = 3.09, *p* = 0.004; Figure 4). Overall, this result is in line with m1, confirming that the stimulus manipulation strongly affects Pre-schoolers rather than School-age children.

Model 3 (m3, Supplementary Table S6) investigated the impact of Group and Stimulus condition on the dependent variable MV, modelled with a Gamma distribution. Results evidenced the sole significant main effect of the type of stimulus on MV, with lower scores in the 2-Dots condition when compared to the Baseline (*b* = −0.10, SE = 0.04, *z* = −2.33, *p* = 0.020). No major statistical effects emerged from Model 4 and Model 5 on the effect of predictors on two temporal parameters, respectively TMDevRight and TMV.

Model diagnostics (DHARMa) indicated a generally satisfying fit for m1 and m2, suggesting in both cases that fixed and random effects contributed meaningfully to the prediction of MH and MDevLeft. For the remaining models (m3, m4, m5), diagnostics revealed some deviation from normality of residual distributions, as evidenced by the significant Kolmogorov–Smirnov test (*p* < 0.001). Limited overdispersion was detected in m4, and within-group deviations from uniformity were also detected in m5. These models explained a modest proportion of the variance, indicating that individual differences accounted for most of the variability. Therefore, it is important to note that the observed effects in the latter models, while statistically reliable, are relatively modest in size and should be generalized with caution.

As a last but important note, MPI scores were available only for school-age participants, as this measure could not be collected in the preschool subsample. As a result, MPI could not be included in the same inferential model together with the developmental grouping predictor, nor tested as a moderator across the full developmental range. To evaluate whether individual differences in MPI could play a relevant role within the subset of School-age children, we conducted additional mixed-effects control analyses restricted to the school-age subsample as a robustness check, testing MPI × condition moderation on the kinematic outcomes. Across all control models (MH, MDevLeft, MV, TMDevRight, and TMV), the interaction was not significant, indicating no evidence that MPI systematically moderated the effect of the experimental condition on kinematic performance within the school-age group. Full results from these control analyses are also reported in the Supplementary material.

## Discussion

Previous kinematic studies suggest that processing numerical magnitudes—both Arabic digits and dot arrays—in the visual domain can influence action execution (Lindemann et al., 2007; Rugani et al., 2017, 2018). However, such effects have been directly explored mainly in adults, and evidence in children remains scarce (Decarli et al., 2022a, 2022b, 2025), leaving open the question of how this association develops across childhood and how it may be reshaped by experience and formal education.

In the present study, we compared the finger–kick responses of preschoolers (4–6 years) and school-aged children (7–11 years) after observing numerical stimuli (2- or 8-Dots arrays) or a non-numerical baseline stimulus (a soccer goal). The task was designed to avoid cognitive biases arising from associations between grasping and physical size, and proved both suitable and engaging for young participants.

The use of a novel motor task minimized the influence of previous experience and automatized motor routines, reducing potential confounds related to familiarity or overlearned strategies. Because such task requires active cognitive engagement and online sensorimotor adaptation, it offers an optimal window into how numerical magnitude shapes action execution during development.

### Preschoolers and the mental number line

Pre-schoolers exhibited greater leftward trajectory deviation after observing small numerosities compared to the baseline condition, indicating that their motor actions were modulated by a spatial–numerical mapping consistent with the Mental Number Line (MNL). Comparable effects have been documented in adults, who showed prolonged leftward deviations after viewing small symbolic numbers (Rugani et al., 2018). A more pronounced leftward tilt in the 2-Dots condition indicates that participants oriented further to the left following the presentation of small numbers compared to large numbers. Comparable findings were obtained using an index-finger pointing paradigm (Fischer, 2003). Subsequent investigations in adults (Ishihara et al., 2006) and in 7-year-old children (Möhring et al., 2019) suggest that this bias may reflect the automatic activation of spatially congruent representations during motor preparation.

Despite differences across studies in age and stimulus type, the influence of numerical magnitude on spatial aspects of action appears robust and can be reliably detected via kinematic parameters such as trajectory deviation—an index known to reflect motor intentions and action planning (Georgiou et al., 2007; Becchio et al., 2008). This suggests that the motor system integrates abstract task-relevant information, including numerical magnitude, into action plans.

Evidence that numerical magnitude affects action selection even in very early infancy further suggests that these effects may arise independently of extensive experience (McCrink and Opfer, 2014; Rugani and De Hevia, 2017). Our findings support this view: the association between numerical magnitude and spatial direction emerges before reading and writing are acquired. Crucially, the present task avoided intrinsic spatial–numerical biases common in manual response paradigms (e.g., left/right key presses). While adult associations between small/large numbers and left/right responses may partly stem from overlearned mappings, the present action-based task—novel for children and identical across conditions—reduces this concern and leverages fine-grained kinematic measures to isolate the contribution of numerical processing.

These results align with ATOM theory (Walsh, 2003), supporting the idea of a shared magnitude-processing system across domains such as number, space, and action. Importantly, our data are consistent with developmental accounts proposing that such common coding frameworks emerge early and may later be refined, reshaped, or reweighted through interaction with symbolic systems and formal education (Decarli et al., 2022a, 2022b, 2025).

### Preschoolers vs. older children and adults: developmental, methodological, and experiential factors

Preschoolers also displayed more efficient movement trajectories after observing smaller numerosities (2-Dots arrays), reflected in lower trajectory height – an index of enhanced sensorimotor integration. Increased trajectory height typically reflects prolonged deceleration phases, which occur when the motor system must satisfy higher precision constraints (Marteniuk et al., 1987). By contrast, adults previously tested with the same task showed improved performance after viewing larger symbolic numbers (8), consistent with overlearned associations linking numerical magnitude to physical weight (Rugani et al., 2018).

Although the same kinematic parameter was sensitive to numerical magnitude in both age groups, the direction of the effect differed: preschoolers optimized movements after smaller non-symbolic numerosities, whereas adults did so after larger symbolic numbers. This developmental dissociation may reflect several, non-mutually exclusive factors.

First, differences in stimulus type (non-symbolic vs. symbolic) may play a role. In adults, non-symbolic stimuli often fail to reliably modulate performance (Rugani et al., 2017), a pattern replicated here in school-aged children, suggesting that non-symbolic effects may diminish or become masked with schooling. Second, general motor maturation is unlikely to fully account for the observed pattern. Kinematic scaling during reaching is broadly comparable between children and adults (Kuhtz-Buschbeck et al., 1998), and our paradigm intentionally excluded grasping components to minimize developmental motor confounds.

Third, and most plausibly, accumulated experience with symbolic numbers and culturally shaped spatial conventions may progressively modulate early non-symbolic spatial–numerical mappings. Children appear to possess an early neurocognitive structure linking numerosities and actions, which becomes increasingly differentiated through maturation and formal education. Toddlers’ spontaneous use of finger counting and pointing to scaffold numerical understanding (Noël, 2005; Lafay et al., 2013; Gunderson et al., 2015; Soylu et al., 2018; Bagnoud et al., 2025; Poletti et al., 2025; Thevenot et al., 2025) likely strengthens number–action associations and supports the acquisition of cardinality and one-to-one correspondence (Gallistel and Gelman, 1992; Butterworth, 1999; Andres et al., 2008). As symbolic number knowledge is acquired, neural activation patterns shift from right-lateralized to more bilateral involvement, including left-hemisphere regions (Salillas et al., 2023; Visibelli et al., 2024). Such reorganization may not eliminate number–action mappings, but may reshape or reweight their expression and accessibility—potentially accounting for the reduced or absent non-symbolic effects observed in older children and adults.

These results should be interpreted cautiously due to the relatively small sample with gender imbalance and high inter-individual variability in numerical abilities, as reflected by the MPI index (Table 1). Furthermore, the baseline stimulus differed in its perceptual and semantic properties from the dot arrays, which might have influenced the responses. However, if this were the case, differences should have emerged for both numerical stimuli. Instead, the fact that only the small stimulus, and not the large one, induced movements that differed from those observed in responding to the baseline stimulus leads us to hypothesize the presence of a genuine numerical effect, rather than an effect driven solely by a semantically or visually different stimulus. Additionally, the visual dimensions of the stimuli were not controlled, thus the observed effects may reflect responses to numerosity-correlated visual information rather than to numerical magnitude. The decision not to equate the total surface area of the stimuli and to adopt a soccer goal as the baseline stimulus was motivated by ecological considerations: during the familiarization phase, the children were told that the dots represented the opposing players they were kicking against, and maintaining a constant dot size—in addition to adopting a soccer goal—supported this narrative. Although these limitations were partly mitigated by precise kinematic measurement and advanced statistical modelling, larger samples and more controlled stimuli will be crucial to deepen understanding of the links between numerical representation, numerical skill, and motor performance, and to inform ongoing theoretical debates.

### Numerical representation: ANS or subitizing?

A further open question concerns whether the cardinality of small sets is represented through the Approximate Number System (ANS; Feigenson et al., 2004) or through a specialized system for small quantities (Fu et al., 2022). Subitizing—the rapid, errorless apprehension of up to four items (Kaufman et al., 1949)—differs from estimation of larger sets, where variability increases proportionally with numerosity underlying small- and large-number processing (Jevons, 1871).

However, this dichotomy is not consistently observed. Several studies report continuous behavioral signatures across small and large numerosities, with no clear boundary between subitizing and estimation (Cantlon and Brannon, 2006; Cordes and Brannon, 2009; Rugani et al., 2013, 2014; Reoyo-Serrano et al., 2025), suggesting that apparent dissociations may depend on task demands, attentional load, or response requirements.

Although the present study focused on non-symbolic numerosities, it was not designed to directly dissociate ANS-based estimation from subitizing mechanisms, and we therefore refrain from making strong claims regarding the specific representational system involved. Nevertheless, the adoption of a kinematic approach provides a complementary perspective by capturing fine-grained spatial–numerical biases as they unfold during action execution, rather than relying exclusively on accuracy or response times. Because the task imposes concurrent cognitive and motor demands, this approach may be particularly sensitive to how attentional and sensorimotor constraints modulate numerical representations across development.

Finally, this framework may also help elucidate the contribution of shared neural substrates. Meta-analytic evidence reveals substantial overlap within parietal regions supporting both symbolic number processing and goal-directed hand actions (Ranzini et al., 2022), consistent with accounts proposing partially shared magnitude and action representations. Such convergence offers a plausible neural basis for the influence of numerical magnitude on motor performance observed in the present and previous studies.

## Conclusion

The present study provides evidence for a direct link between numerical processing and motor execution in preschool children, extending previous kinematic findings obtained in adults to an earlier developmental stage. Observing small non-symbolic numerosities enhanced motor efficiency relative to a non-numerical baseline and induced greater leftward trajectory deviations, consistent with an early-emerging spatial–numerical mapping aligned with the Mental Number Line.

This developmental pattern contrasts with adult data, in which larger symbolic numbers facilitate motor performance. Such differences are unlikely to reflect general motor maturation, given the relative stability of basic kinematic parameters across development. Instead, they are more plausibly explained by developmental and educational factors, including the transition from non-symbolic to symbolic numerical representations and increasing exposure to culturally shaped sensorimotor–numerical associations.

Overall, these findings underscore the importance of investigating number–action interactions during early development using tasks that minimize interference from overlearned sensorimotor routines. By revealing how numerical magnitude can directly shape action execution before formal schooling, the present work highlights the potential of embodied and action-based approaches for advancing theoretical models of numerical cognition and for informing educational practices aimed at supporting the acquisition of symbolic numerical skills.

## Data Availability

The datasets presented in this study can be found in online repositories. The online link for the repository is: https://osf.io/unf9y/overview?view_only=780672a28ec947ba9fa469308c425ace.
