# Supplementary material for: Kicking the mental number line: a kinematic investigation of numerical processing in childhood
Source: Front Psychol. 2026 Feb 25;17:1765058. doi: 10.3389/fpsyg.2026.1765058 (PMC12975588; doi:10.3389/fpsyg.2026.1765058)
Supplement: Supplementary file 1 [file Table_1.DOCX]

**Supplementary Material**

**Table S1.** Math tasks.

| **Enumeration Time**  (ET, the total time, 20’’, minus the time taken to pronounce the entire list without errors) | **Additive Calculation Ability**  (ACA, the number of additions performed correctly in 30’’) | **Subtractive Calculation Ability** (SCA, the number of subtractions performed correctly in 30’’) |
| --- | --- | --- |
| 1 | 1+3 = | 4-1 = |
| 2 | 2+2 = | 5-2 = |
| 3 | 5+4 = | 8-1= |
| 4 | 6+2 = | 7-4 = |
| 5 | 9+1 = | 9-5 = |
| 6 | 3+6 = | 4-3 = |
| 7 | 2+7 = | 8-4 = |
| 8 | 4+4 = | 2-0 = |
| 9 | 3+0 = | 9-7 = |
| 10 | 7+1 = | 7-2 = |
| 11 | 9+4 = | 8-6 = |
| 12 | 7+5 = | 5-4 = |
| 13 | 0+7 = | 6-2 = |
| 14 | 6+8 = | 9-8 = |
| 15 | 2+9 = | 2-2= |
| 16 | 6+6 = | 8-3 = |
| 17 | 4+3 = | 3-2 = |
| 18 | 7+9 = | 7-3 = |
| 19 | 8+4 = | 9-7 = |
| 20 | 5+2 = | 4-4 = |

**Table S2.** Stimuli parameters (in px) for numerosities 2 and 8 generated by GeNEsIS software.

| **Numerosity** | **Convex Hull** | **Density** | **Inter Distance** | **Total  Area** | **Total Perimeter** | **Radius minimum** | **Radious maximum** |
| --- | --- | --- | --- | --- | --- | --- | --- |
| 2 | 31.04 | 0.06 | 15.83 | 5.09 | 11.31 | 0.9 | 0.9 |
| 8 | 135.73 | 0.06 | 8.69 | 20.36 | 45.24 | 0.9 | 0.9 |


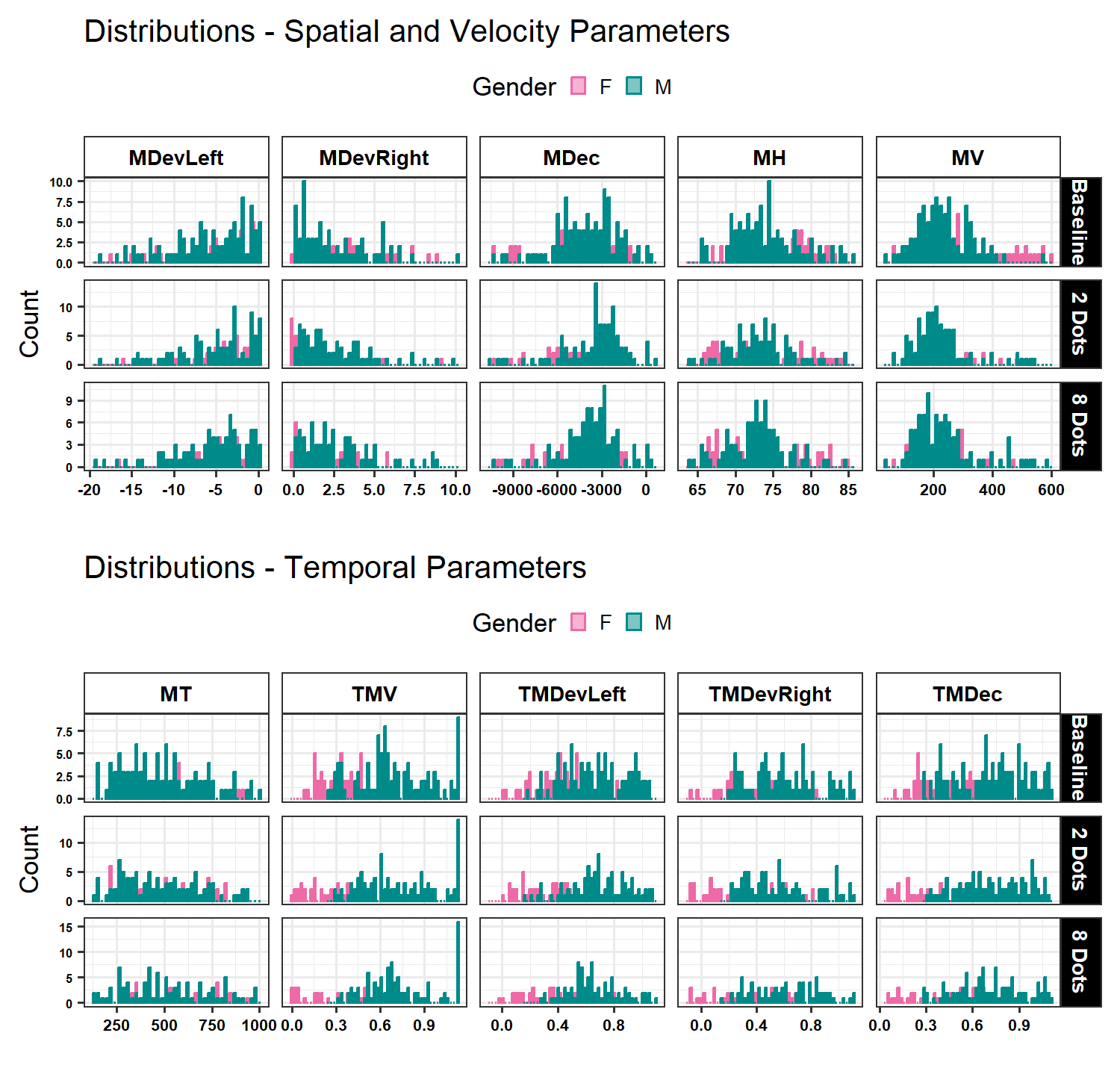


**Figure S1.** Distributions of spatial, velocity, and temporal parameters across genders. Histograms show the distribution of parameter values for Pre-school Age (orange) and School Age (green) participants, separately for conditions (vertical facets, columns) and type of stimulus (Baseline, 2-Dots and 8-Dots; horizontal facets, rows). Parameters are grouped into Spatial and Velocity Parameters (top panel: MDevLeft, MDevRight, Mdec, MH, MV) and Temporal Parameters (bottom panel: MT, TMV, TMDevLeft, TMDevRight, TMDec).

**Table S3.** Kinematic parameters (mean within-participant average and standard deviation). These values are obtained by averaging individual, per each participant, individual mean and SD scores across Condition and Kinematic parameters. The resulting values were averaged on the full sample, so as to obtain a mean within-participant average and SD scores as an additional summary statistics of intrapersonal variance for clarifying reliability and consistency of individual performances.

|  | **2-Dots** | **8-Dots** | **Baseline** |
| --- | --- | --- | --- |
| **MH** | 73.552±2.198 | 73.721±2.083 | 74.740±2.130 |
| **MDevLeft** | -5.208±3.014 | -5.678±3.248 | -5.730±3.315 |
| **MDevRight** | 2.318±1.530 | 2.613±1.581 | 2.487±1.784 |
| **MV** | 236.250±84.100 | 237.697±70.431 | 261.088±74.557 |
| **MDec** | 4096.349±1701.056 | 4250.569±1577.751 | 4413.991±1769.641 |
| **MT** | 494.387±210.510 | 539.129±173.340 | 487.099±178.812 |
| **TMV** | 0.552±0.180 | 0.581±0.185 | 0.540±0.181 |
| **TMDec** | 0.594±0.177 | 0.591±0.189 | 0.600±0.176 |
| **TMDevRight** | 0.459±0.254 | 0.504±0.209 | 0.477±0.217 |
| **TMDevLeft** | 0.524±0.182 | 0.545±0.175 | 0.546±0.179 |

**Table S4*.*** Generalized Mixed Effect Model (GLMMs) summary studying the interactional role of Group (Pre-School, School Age) and Type of Stimulus (2-Dots, 8-Dots, Baseline) on the actuation of MH kinematic measure. The t-student family distribution was imposed to the model.

| **Model 1: MH (t-student distribution)** | | | | | |
| --- | --- | --- | --- | --- | --- |
| ***Predictors*** | ***Estimates*** | ***SE*** | ***95% CI*** | ***z*** | ***p*** |
| (Intercept) | 69.42 | 1.14 | [67.18, 71.66] | 60.85 | **<.001** |
| Group [School] | 6.58 | 1.40 | [3.84, 9.32] | 4.70 | **<.001** |
| Stimulus [Baseline] | -1.11 | 0.18 | [-1.47, -0.75] | -6.01 | **<.001** |
| Stimulus [8 Dots] | 0.50 | 0.16 | [0.18, 0.81] | 3.09 | **.002** |
| Group [School] 🞪 Stimulus [Baseline] | 0.82 | 0.22 | [0.38, 1.26] | 3.65 | **<.001** |
| Group [School] 🞪 Stimulus [8 Dots] | -0.33 | 0.21 | [-0.74, 0.08] | -1.57 | .115 |
| N = 21; Observations = 535; τ_00_ = 8.94; ICC: 0.88; σ^2^ = 1.28; R2 (marginal/conditional): 0.494/0.937 | | | | | |

**Table S5.** Generalized Mixed Effect Model (GLMMs) summary studying the interactional role of Group (Pre-School, School Age) and Type of Stimulus (2-Dots, 8-Dots, Baseline) on the actuation of MDevLeft kinematic measure. The t-student family distribution was imposed to the model.

| **Model 2: MDevLeft (t-student distribution)** | | | | | |
| --- | --- | --- | --- | --- | --- |
| ***Predictors*** | ***Estimates*** | ***SE*** | ***95% CI*** | ***z*** | ***p*** |
| (Intercept) | -6.71 | 0.93 | [-8.58, -4.88] | -7.19 | **< .001** |
| Group [School] | 2.98 | 1.13 | [0.76, 5.20] | 2.64 | **.008** |
| Stimulus [Baseline] | 1.40 | 0.45 | [0.52, 2.28] | 3.13 | **.002** |
| Stimulus [8 Dots] | -0.56 | 0.42 | [1-.39, 0.27] | -1.33 | .185 |
| Group [School] 🞪 Stimulus [Baseline] | -1.39 | 0.50 | [-2.37, -0.42] | -2.79 | **.005** |
| Group [School] 🞪 Stimulus [8 Dots] | 0.81 | 0.47 | [-0.12, 1.74] | 1.71 | .088 |
| N = 21; Observations = 470; τ_00_ = 5.42; ICC: 0.69; σ^2^ = 2.40; R2 (marginal/conditional): 0.218/0.760 | | | | | |

**Table S6.** Generalized Mixed Effect Model (GLMMs) summary studying the interactional role of Group (Pre-School, School Age) and Type of Stimulus (2-Dots, 8-Dots, Baseline) on the actuation of MV kinematic measure. The Gamma family distribution was imposed to the model.

| **Model 3: MV (Gamma distribution)** | | | | | |
| --- | --- | --- | --- | --- | --- |
| ***Predictors*** | ***Estimates*** | ***SE*** | ***95% CI*** | ***z*** | ***p*** |
| (Intercept) | 5.54 | 0.08 | [5.38, 5.69] | 70.23 | **.000** |
| Group [School] | -0.14 | 0.10 | [-0.33, 0.05] | -1.43 | .152 |
| Stimulus [Baseline] | -0.10 | 0.04 | [-0.18, -0.02] | -2.33 | **.020** |
| Stimulus [8 Dots] | 0.03 | 0.04 | [-0.05, 0.11] | 0.78 | .435 |
| Group [School] 🞪 Stimulus [Baseline] | 0.04 | 0.05 | [-0.05, 0.14] | 0.91 | .365 |
| Group [School] 🞪 Stimulus [8 Dots] | -0.02 | 0.05 | [-0.12, 0.08] | -0.44 | .662 |
| N = 21; Observations = 553; τ_00_ = 0.04; ICC: 0.22; σ^2^ = 0.13; Delta R2 (marginal/conditional): 0.038/0.253 | | | | | |
